# Prevalence of Antibodies to Zika Virus in Mothers from Hawaii Who Delivered Babies with and without Microcephaly between 2009-2012

**DOI:** 10.1371/journal.pntd.0005262

**Published:** 2016-12-20

**Authors:** Mukesh Kumar, Lauren Ching, Joshua Astern, Eunjung Lim, Alexander J. Stokes, Marian Melish, Vivek R. Nerurkar

**Affiliations:** 1 Department of Tropical Medicine, Medical Microbiology and Pharmacology, John A. Burns School of Medicine, University of Hawaii at Manoa, Honolulu, Hawaii United States of America; 2 Pacific Center for Emerging Infectious Diseases Research, John A. Burns School of Medicine, University of Hawaii at Manoa, Honolulu, Hawaii United States of America; 3 Department of Cell and Molecular Biology, John A. Burns School of Medicine, University of Hawaii at Manoa, Honolulu, Hawaii United States of America; 4 Office of Biostatistics and Quantitative Health Sciences, Department of Tropical Medicine, Medical Microbiology and Pharmacology, John A. Burns School of Medicine, University of Hawaii at Manoa, Honolulu, Hawaii United States of America; 5 Department of Pediatrics, John A. Burns School of Medicine, University of Hawaii at Manoa, Honolulu, Hawaii United States of America; University of Rhode Island, UNITED STATES

## Abstract

Zika virus (ZIKV) is an emerging mosquito-borne pathogen. ZIKV infection is linked to the development of severe fetal abnormalities that include spontaneous abortion, stillbirth, hydranencephaly, and microcephaly. ZIKV outbreaks have been recorded in the United States. We recently demonstrated the first congenital ZIKV infection in the United States. In this study, we investigated archived blood samples from six mothers who gave birth to babies with microcephaly and 12 mothers who gave birth to healthy babies in Hawaii between 2009 and 2012. We tested maternal blood for the presence of ZIKV IgM and IgG antibodies using commercially available human ZIKV IgM and IgG ELISA kits. Blood from one mother who delivered babies with microcephaly tested positive for ZIKV IgM antibody (16.6%) and blood from three mothers tested positive for ZIKV IgG antibody (50%). ZIKV showed a trend toward significance with microcephaly. ZIKV IgG antibody positive mothers were more likely to deliver babies with microcephaly than mothers who were negative for ZIKV IgG antibodies (Odds ratio [OR] = 11.0, 95% confidence interval [CI] = 0.8–147.9, p = 0.083). Similarly, ZIKV IgM antibody positive mothers were also more likely to deliver babies with microcephaly than mothers who were negative for ZIKV IgM antibody (OR = 6.8, 95% CI = 0.2–195.1). These data provide further evidence of a link between ZIKV infection and microcephaly and suggests presence of ZIKV positive cases and associated microcephaly in the United States as early as 2009.

## Author Summary

Zika virus (ZIKV) infection is linked to the development of severe fetal abnormalities that include spontaneous abortion, stillbirth, hydranencephaly, and microcephaly. The WHO in early February 2016 declared global ZIKV outbreaks and its link to birth defects an international public health emergency. ZIKV outbreaks have been recorded in the United States. In a quest to find a link between ZIKV infection and babies born with microcephaly in Hawaii, we investigated archived blood samples from mothers who gave birth in Hawaii between 2009 and 2012 to babies with microcephaly. ZIKV antibodies were detected in three (50%) out of total six mothers who delivered babies with microcephaly. ZIKV showed a trend toward significance with microcephaly. This report adds to the potential evidence of a link between ZIKV infection and microcephaly and suggests presence of ZIKV positive cases and associated microcephaly in the United States as early as 2009.

## Introduction

Zika virus (ZIKV) is an emerging mosquito-borne pathogen that is part of the Spondweni serocomplex of the genus *Flavivirus*, family *Flaviviridae*. Mosquitoes of the *Aedes* genus transmit ZIKV. Approximately 80% of individuals infected with ZIKV have no symptoms [1, 2]. ZIKV caused only sporadic cases of infection in Africa and Southeast Asia until 2007, when the first large outbreak occurred in the Yap State in Micronesia [3, 4]. Another outbreak in French Polynesia in 2013 was notable for being associated with an increase in cases of Guillain-Barré syndrome (GBS) [5–7]. In 2015, the virus was first reported in Brazil and since then has spread through several additional countries in South and Central America and the Caribbean. Simultaneously, several of these countries have seen a dramatic increase in the incidence of infants born with microcephaly [1, 2, 8]. Since then ZIKV outbreaks have been recorded in the United States and Hawaii has encountered few cases of travel related ZIKV [9–11]. Similarly, since fall 2015 Puerto Rico has seen a sudden increase in cases of ZIKV infection particularly in pregnant women [12]. During the current epidemic in Latin America, ZIKV infection has been linked to the development of severe fetal abnormalities that include spontaneous abortion, stillbirth, hydranencephaly, microcephaly, and placental insufficiency that may cause intrauterine growth restriction [1, 2, 8]. The rapid spread of ZIKV through the Americas, together with the association of infection with microcephaly and GBS, has resulted in the World Health Organization declaring a public health emergency. No effective therapies currently exist for treating patients infected with ZIKV.

We recently demonstrated the first congenital ZIKV infected case in the United States, confirmed by high ZIKV IgM antibody titers in serum and cerebrospinal fluid [10]. In this case, a ZIKV-infected mother delivered a baby with microcephaly. In a quest to find a link between ZIKV infection and babies born with microcephaly, we investigated archived blood samples from mothers who gave birth in Hawaii between 2009 and 2012 to babies with microcephaly. We tested maternal blood for the presence of ZIKV IgM and IgG antibodies using commercially available human ZIKV IgM and IgG ELISA kits [11].

## Materials and Methods

### Ethics statement

Ethical approval for this study was obtained from the Institutional Review Board of the University of Hawaii (CHS#23889). All samples were collected with post-partum written informed consent.

### Human plasma samples

Patient information and plasma samples were obtained from the University of Hawaii Biorepository (UHB). The UHB archived plasma samples from mothers who gave birth at the Kapiolani Medical Center for Women and Children (KMCWC) in Hawaii between 2007 and 2013 with post-partum written informed consent. All the samples were collected post-partum. In years 2007 and 2008 we did not find in the database mothers who gave birth to babies with microcephaly. However, from 2009 onwards, we identified six mothers who gave birth to babies with microcephaly.

### Microcephaly diagnosis

The attending physicians identified all cases of microcephaly. Additionally, retrospectively pediatrician MM, coauthor on this publication, reanalyzed the clinical data (head circumference, body weight, chest circumference and body length) for all babies along with mothers’ gestational age. Microcephaly was defined as head circumference < 2 standard deviations from the mean or < 3rd percentile using Fenton Head Circumference Charts. MM was blinded to mothers ZIKV serological results.

### Selection of controls

Controls were selected from the plasma samples stored in the UHB. We selected 12 mothers who gave birth to healthy babies during the same timeframe in which we identified six mothers who gave birth to babies with microcephaly (2009–2012), using a 1:2 ratio to match with the cases. Controls were selected based on mothers age and gestational age. There was no significant difference between the mothers age and mean gestational age for both sets of mothers who gave birth to healthy babies or babies with microcephaly. We also attempted to match the controls using ethnicity. For both set of mothers ethnicity was 100% Asians or mixed Asians (mixed with Pacific Islanders including Native Hawaiians) (Table 1).

**Table 1:** Clinical and epidemiological characteristics of mothers who gave birth in Hawaii (2009–2012)

ID	Age	Gestation Age (Weeks)	Delivery year	Ethnicity	Microcephalus Baby	ZIKV IgG		ZIKV IgM
						RU/mL	+/-	
1	28	38	2011	Asian	Yes	118	**+**	**+**
2	21	37	2009	Asian	Yes	23	**+**	**-**
3	32	37	2009	Asian	Yes	19	**+**	**-**
4	33	39	2009	Mixed Asian and Pacific Islander	Yes	6	**-**	**-**
5	22	40	2010	Mixed Asian and Pacific Islander	Yes	≤2	**-**	**-**
6	21	39	2010	Mixed Asian and Pacific Islander	Yes	≤2	**-**	**-**
7	27	37	2010	Asian	No	40	**+**	**-**
8	21	37	2010	Asian	No	15	**-**	**-**
9	27	38	2009	Asian	No	7	**-**	**-**
10	33	39	2012	Mixed Asian and Pacific Islander	No	≤2	**-**	**-**
11	32	38	2010	Asian	No	≤2	**-**	**-**
12	22	40	2012	Mixed Asian and Pacific Islander	No	≤2	**-**	**-**
13	32	38	2010	Asian	No	≤2	**-**	**-**
14	32	35	2011	Mixed Asian and Pacific Islander	No	≤2	**-**	**-**
15	28	38	2012	Asian	No	≤2	**-**	**-**
16	35	39	2012	Mixed Asian and Pacific Islander	No	≤2	**-**	**-**
17	23	38	2012	Asian	No	≤2	**-**	**-**
18	30	40	2013	Asian	No	≤2	**-**	**-**

ZIKV; Zika virus

### ZIKV ELISA

ZIKV-specific IgM and IgG antibodies were determined in the samples using EUROIMMUN anti-ZIKV IgM and IgG ELISA, respectively, as per manufacturers’ instructions [13]. Briefly, plasma samples were diluted 1:101 and incubated in the wells coated with recombinant non-structural protein (NS1) of ZIKV. To detect the bound antibodies, a second incubation was conducted using an enzyme-labeled anti-human IgM or anti-human IgG (enzyme conjugate) catalyzing a color reaction. Before IgM detection, samples were pre-incubated with sample buffer containing rheumatoid factor absorbent as recommended. Photometric measurement of the color intensity was determined at a wavelength of 450 nm and a reference wavelength between 620 and 650 nm as per manufacturers’ instruction using a Victor 3 microtiter reader (Perkin Elmer).

ELISA results were interpreted as per EUROIMMUN recommendations [13]. For IgG ELISA, a standard curve was obtained by point-to-point plotting of the extinction values measured for the three calibration sera against corresponding units. As no quantitated international reference serum exists for antibodies against ZIKV, the calibration is performed in relative units (RU). The standard curve was used for the determination of the antibody concentration in samples. IgG values above 22 RU/mL were considered positive. IgG values between 16 to 22 RU/mL were considered borderline positive and below 16 RU/mL were considered negative.

For IgM ELISA, results were evaluated semi-quantitatively by calculating a ratio of the extinction value of the sample over the extinction value of the calibrator. A ratio of more than 1.1 was considered positive. A ratio between 0.8 and 1.1 was considered borderline positive and below 0.8 was considered negative. For every group of tests conducted, the extinction values of the calibrator and the relative units and ratios determined for the positive and negative controls for both IgG and IgM ELISA were within the limits stated for the relevant test kit lot.

### Statistical analysis

Fisher’s exact tests for categorical variables and two-sample t tests for continuous variables were used to compare between normal babies and babies with microcephaly. Odds ratios (ORs) and 95% confidence intervals (CIs) were also computed to investigate association between microcephaly and subject characteristics. A correction of 0.5 was used to compute OR if a cell contains zero. P-value <0.05 was considered statistically significant and p-value <0.10 was considered trend toward significance.

## Results and Discussion

An overview of clinical and epidemiological data from the 18 cases is presented in Table 1. There was no significant difference between controls and cases on the matching factors (Table 2). The mean mothers age was 28 years (range 21–35) with no significant difference between mothers who gave birth to healthy babies vs. babies with microcephaly; 29 vs. 26 years. Mean gestational age was 38 weeks (range 35–40) for both sets of mothers who gave birth to healthy babies or babies with microcephaly. Of the 18 mothers, six gave birth to babies with microcephaly. Blood from one mother who delivered a baby with microcephaly tested positive for ZIKV IgM antibody (16.6%) and blood from three mothers tested positive for ZIKV IgG antibody (50%). ZIKV IgG antibody was detected in one of 12 (8.3%) mothers who delivered healthy babies and all 12 mothers were negative for ZIKV IgM antibodies. ZIKV showed a trend toward significance with microcephaly. ZIKV IgG antibody positive mothers were more likely to deliver babies with microcephaly than mothers who were negative for ZIKV IgG antibodies (OR = 11.0, 95% CI = 0.8–147.9, p = 0.083). Similarly, ZIKV IgM antibody positive mothers were also more likely to deliver babies with microcephaly than mothers who were negative for ZIKV IgM antibody (OR = 6.8, 95% CI = 0.2–195.1, p = 0.333) (Table 2). Of the three ZIKV IgG positive mothers (one was borderline positive) who gave birth to babies with microcephaly, one was ZIKV IgM positive and other two were IgM negative. Interpretation of the two ZIKV IgG positive and IgM negative result is challenging. One possible explanation is that IgM was positive in the mothers’ blood earlier in gestation. Propensity score was also estimated using a logistic regression with the matching factors (i.e., mothers’ age, gestational age, ethnicity). There was no significant difference between two groups in propensity score (control: 0.31±0.12 vs. case: 0.39±0.18, p = 0.34 by nonparametric test). The ORs adjusting for the propensity score were similar to the unadjusted ORs.

**Table 2:** Bivariate Association between Microcephaly and Subject Characteristic

	Microcephaly, n (%)		p-value	Odds Ratio (95% CI)
Variable	No (n = 12)	Yes (n = 6)		
Mother’s Age (year), Mean ± SD	29 ± 5	26 ± 6	0.337	0.9 (0.7–1.1)
Gestational Age (week), Mean ± SD	38 ± 1	38 ± 1	0.692	1.2 (0.5–2.6)
Gestational Age			1.000	
Preterm (≤37 weeks)	3 (25.0%)	2 (33.3%)		1.5 (0.2–12.8)
Normal (38–42 weeks)	9 (75.0%)	4 (66.7%)		Reference
Zika virus IgG			0.083	
Present	1 (8.3%)	3 (50%)		11.0 (0.8–147.9)
Absent	11 (91.7%)	3 (50%)		Reference
Zika virus IgM			0.333	
Present	0 (0%)	1 (16.7%)		6.8 (0.2–195.1)
Absent	12 (100%)	5 (83.3%)		Reference

Column percentage. CI = confidence interval. SD = standard deviation.

Note: p-value was obtained by Fisher’s exact test for categorical variable or two sample t test for continuous variable. A correction of 0.5 was used to compute odds ratio in every cell that contains a zero.

Laboratory results for chlamydia, hepatitis B virus, and gonococcus were available for 18 mothers and all were negative. All three ZIKV positive mothers who gave birth to babies with microcephaly were tested negative for chlamydia, hepatitis B virus, and gonococcus. Similarly, laboratory results for syphilis tests were available for all six mothers who gave birth to babies with microcephaly and all were non-reactive. However, two mothers who were positive for herpes simplex virus, of which, one gave birth to a baby with microcephaly and both mothers were negative for ZIKV IgG and IgM antibodies.

Diagnosis of ZIKV infection has been complicated by cross-reactivity between antibodies to other flaviviruses and by the fact that dengue virus is endemic in the Pacific [1]. The high degree of cross-reactivity of currently available serological flavivirus assays is a major issue of concern. Therefore, in this study we employed EUROIMMUN anti-ZIKV IgM and anti-ZIKV IgG ELISA based on ZIKV NS1 antigen. It has been demonstrated that the Euroimmun ELISA is highly specific and reliable when used for patients with previous flavivirus exposure or vaccination. In one published study, none of the samples from patients with tick borne encephalitis virus, dengue virus, and West Nile virus infection and recent yellow fever vaccination demonstrated reactivity above the threshold for positivity, demonstrating the high specificity of the Euroimmun ZIKV IgM and IgG ELISA [13]. Similarly, we also did not observe any reactivity above threshold for a confirmed dengue virus positive and ZIKV negative sample using this kit. Moreover, we recently reported the first mother in the United States who gave birth to a baby with microcephaly, who was positive for ZIKV by plaque reduction neutralization test [10].

Outbreaks of ZIKV infection have occurred in Southeast Asia, and the Pacific Islands and the virus is endemic in regions of Africa. Currently, there is an ongoing outbreak in the Americas and autochthonous cases have been reported from 37 countries and territories worldwide [1, 7, 8]. ZIKV outbreaks have been recorded in the United States [9, 11, 14]. To date, there have been no cases of locally acquired ZIKV infection in Hawaii. Several cases of travel related ZIKV infection have been reported by the Hawaii Department of Health over the past 5 years. However, travel history for these archived samples is not available. In the Pacific ZIKV outbreak was first reported in 2007 on the Island of Yap, Federated States of Micronesia (FSM), followed by widespread small outbreaks throughout the Pacific [3–7]. The period of the outbreak (2007 to now) overlaps with collection of samples analyzed in this study (2009 to 2012).

As mentioned above, except for travel related ZIKV cases ZIKV outbreak has not occurred in Hawaii. We therefore hypothesize that pregnant women may have been exposed to ZIKV during their visit to Pacific Island Nations resulting in babies born with microcephaly. Due to close proximity and historic ties between the islands, travel to various islands in the Pacific from Hawaii is relatively frequent and the duration of stay is comparatively long. The majority of travelers from Hawaii to these islands are visiting friends and relatives (VFR). International studies suggest that these travelers experience a higher risk of contracting travel-related infectious diseases compared to other groups of international travelers [15]. VFR travelers are less likely to obtain pre-travel medical advice, usually have closer contact with local populations and their associated housing conditions, and are more likely to have a longer duration of travel. Interestingly, majority (5 out of 6) microcephalus babies reported in this study were delivered in the years 2009–2010 (Table 1), which coincides with ZIKV outbreaks in the Pacific starting in 2007 [3–7]. Moreover, one baby with microcephaly delivered in the year 2015 in Hawaii [10] was associated with ZIKV outbreak in Latin America [1, 8]. This data collectively suggest increase in microcephaly cases in Hawaii coincide with ZIKV outbreak in the Pacific and Latin America.

Based on Hawaii Birth Defects Surveillance Report (1986–2005), there was a declining trend of incidence of microcephaly in Hawaii, with a rate of 13.6 per 10,000 total births in 1986 to 4.8 per 10,000 total births in 2005. Over the period (1986–2005), a total of 370 cases of microcephaly were reported in Hawaii, which is equivalent to 9.4 per 10,000 total births [16]. However, based on the University of Hawaii Biorepository data, over the period of 2007–2013, microcephaly rate was 14.7 per 10,000 total births. This increase in microcephaly rate coincides with ZIKV outbreaks in the Pacific starting in 2007 [3–7].

Limitation of this study is the lack of ZIKV plaque reduction neutralization test and PCR testing for detection of viral RNA due to insufficient quantity of plasma. However, recently published report using EUROIMMUN ZIKV ELISA gives credence to our study [13]. In this retrospective study, our analysis was restricted to the link between ZIKV and microcephaly. It will be important to ascertain whether ZIKV is associated with other fetal or neonatal neurological complications as suggested by other investigators [17]. Our study was retrospective, with a small sample size and prospective studies in a large scale to assess incidence of ZIKV-associated microcephaly in Hawaii and other regions of the United States are urgently needed.

In this study, we investigated the association between ZIKV and microcephaly. Due to small sample size, the association did not reach the common statistical significance of p-value 0.05. However, this report adds to the potential evidence of a link between ZIKV infection and microcephaly and suggests presence of ZIKV positive cases and associated microcephaly in the United States as early as 2009.

## Supporting Information

S1 Checklist STROBE Checklist. (DOC)
